# Prognostic significance of asymmetric dimethyl arginine level in pediatric patients with COVID-19 infection and MIS-C

**DOI:** 10.1007/s00431-025-06079-8

**Published:** 2025-03-12

**Authors:** Memduha Sari, Fatih Akin, Abdullah Yazar, Ahmet Osman Kilic, Ozge Metin Akcan, Abdullah Akkus, Mehmet Uyar, Cemile Topcu, Mustafa Genceli

**Affiliations:** 1 Department of Pediatrics, Mut State Hospital, 33600 Mersin, Turkey; 2 Department of Pediatrics, Faculty of Medicine Hospital, Necmettin Erbakan University, Konya, Turkey; 3 Departments of Pediatric Infectious Diseases, Faculty of Medicine Hospital, Necmettin Erbakan University, Konya, Turkey; 4 Department of Public Health, Faculty of Medicine Hospital, Necmettin Erbakan University, Konya, Turkey; 5 Department of Biochemistry, Faculty of Medicine Hospital, Necmettin Erbakan University, Konya, Turkey

**Keywords:** MIS-C, ADMA, COVID-19, Endothelial damage, Children

## Abstract

Coronavirus disease 2019 (COVID-19) and COVID-19-related multisystem inflammatory syndrome in children (MIS-C) is known to be a life-threatening health problem worldwide. The study investigates the potential relationship between asymmetric dimethylarginine (ADMA) levels and disease severity in such conditions. We conducted an observational, prospective study between July 2021 and January 2022. The study enrolled 98 patients diagnosed with COVID-19, 21 patients diagnosed with MIS-C, and 42 healthy individuals who served as a control group. The COVID-19 patients were further categorized into three subgroups based on their level of care: outpatients, those requiring hospitalization, and those requiring intensive care. The MIS-C patients formed a distinct fourth group. COVID-19 outpatients had a median ADMA level of 8097.0 ng/L (interquartile range: 6436.06–10840.0 ng/L), while those requiring hospitalization had a higher level of 13,195.60 ng/L (11,472.4–15,862.2 ng/L). Patients in intensive care exhibited the highest median ADMA level at 19,361.4 ng/L (15,596.65–23,367.9 ng/L). MIS-C patients also had elevated ADMA levels, with a median of 15,735.50 ng/L (13,486.6–20,532.5 ng/L). Receiver operating characteristic (ROC) curve analysis revealed that an ADMA level of 6135.15 ng/L could distinguish between patients and controls with 95% sensitivity, 100% specificity, 100% positive predictive value, and 87.5% negative predictive value.

*Conclusions*: In conclusion, our study is the first to investigate ADMA levels in children with COVID-19 and MIS-C. We found that ADMA levels were significantly elevated in children with COVID-19 requiring intensive care and those with MIS-C, suggesting a potential role for ADMA as a biomarker of endothelial dysfunction in these populations.

**Table:** 

**What Is Known:** *• Endothelial dysfunction is a determinant of poor prognosis in various cardiovascular diseases and plays a critical role in the pathogenesis of COVID-19 and MIS-C.* *• Asymmetric dimethylarginine (ADMA) is a well-known biomarker of endothelial dysfunction. Elevated levels of ADMA adversely affect vascular endothelial function by reducing nitric oxide production.* **What Is New:** *• It is the first to show that elevated ADMA levels in children with COVID-19 and MIS-C are associated with disease severity.* *• ADMA has been identified as a potential biomarker that can be used to assess the prognosis of COVID-19 and MIS-C in children and to predict the severity of the disease.*

**What Is Known:**

*• Endothelial dysfunction is a determinant of poor prognosis in various cardiovascular diseases and plays a critical role in the pathogenesis of COVID-19 and MIS-C.*

*• Asymmetric dimethylarginine (ADMA) is a well-known biomarker of endothelial dysfunction. Elevated levels of ADMA adversely affect vascular endothelial function by reducing nitric oxide production.*

**What Is New:**

*• It is the first to show that elevated ADMA levels in children with COVID-19 and MIS-C are associated with disease severity.*

*• ADMA has been identified as a potential biomarker that can be used to assess the prognosis of COVID-19 and MIS-C in children and to predict the severity of the disease.*

## Introduction

The COVID-19 pandemic has profoundly impacted global health, causing significant morbidity and mortality. While children generally experience mild or asymptomatic infections, severe cases and deaths have been reported [1]. A unique challenge of this pandemic has been the emergence of Multisystem Inflammatory Syndrome in Children (MIS-C), a hyperinflammatory disease associated with COVID-19. MIS-C is characterized by uncontrolled systemic inflammation, fever, hypotension, and cardiac dysfunction [2, 3].

Endothelial dysfunction, an early hallmark of various cardiovascular diseases and a predictor of poor outcomes may play a pivotal role in the pathogenesis of COVID-19 and MIS-C. Endothelial health is closely linked to nitric oxide (NO) production, which regulates vascular tone, structure, and cell–cell interactions within blood vessels. As a potent vasodilator, inhibitor of smooth muscle cell proliferation, and modulator of platelet and monocyte adhesion, NO is considered an anti-atherogenic molecule [4]. Emerging evidence suggests that endothelial dysfunction is a key factor in the pathophysiology of viral infections, including COVID-19 [5–7]. Understanding this link is crucial for comprehending the multisystemic effects of these viruses and developing appropriate therapeutic strategies.

Asymmetric dimethylarginine (ADMA), an endogenous inhibitor of nitric oxide synthase (NOS), is synthesized in endothelial cells and found in various bodily fluids and tissues. ADMA impairs vascular NO production, leading to impaired endothelium-dependent vasodilation [8]. This is significant because angiotensin-converting enzyme 2 (ACE2), a key regulator of NO release, is also the host cell receptor for severe acute respiratory syndrome coronavirus type 2 (SARS-CoV-2), the virus responsible for COVID-19. Thus, a connection exists between COVID-19 infection and endothelial NO signaling [9].

The pathogenesis of COVID-19 involves diffuse alveolar damage, inflammatory infiltrates, endotheliitis, progressive respiratory failure, coagulopathy, microthrombi formation, and multi-organ dysfunction. Endothelial cells are thought to be central to this process [10]. Given the importance of NO for endothelial function and ADMA’s role in NO production, ADMA may have clinical significance in COVID-19.

ADMA and its counterpart, symmetric dimethylarginine (SDMA), are endogenous modulators of NO synthesis. Studies have implicated them in the pathogenesis of various conditions, including endothelial dysfunction, atherosclerosis, oxidative stress, inflammation, uremia, apoptosis, autophagy, and impaired immune function [11–17].

While research on serum ADMA levels in adult COVID-19 patients is limited, our study aims to explore the potential of ADMA as a biomarker for predicting the prognosis and clinical course of COVID-19 and MIS-C in children. We hypothesize that ADMA levels may be indicative of disease severity and outcomes in these pediatric populations.

## Materials and methods

Our observational, prospective study, conducted between July 2021 and January 2022, included 98 children diagnosed with COVID-19 (confirmed by polymerase chain reaction), 21 children diagnosed with MIS-C (meeting CDC diagnostic criteria) [18], and 42 age-matched healthy children serving as a control group. The control group consisted of children without any underlying disease who had blood samples collected for unrelated reasons. The COVID-19 patients were further categorized into three subgroups based on their level of care: outpatients, those requiring hospitalization in a general ward, and those requiring intensive care. The MIS-C patients formed a separate group. Importantly, individuals who had received a COVID-19 vaccine were excluded from both the patient and control groups due to the potential for vaccination to influence ADMA levels.

Remaining serum was collected from the initial blood samples of both the patient and control groups by centrifugation at 3500 RPM for 5 min. The serum was then stored in Eppendorf tubes at −80 °C until analysis. All samples were collected within a 6-month timeframe. On the day of analysis, samples were thawed to room temperature (15–18 °C), homogenized, and subsequently measured.

Serum ADMA levels were measured using a commercially available 96-well enzyme-linked immunosorbent assay (ELISA) kit (BTLAB, catalog number E1887Hu). The assay was performed at the Medical Biochemistry Research Laboratory of Necmettin Erbakan University Meram Medical Faculty. The kit’s measurement range was 200–60,000 ng/L with a sensitivity of 100.21 ng/L and an intra-assay coefficient of variation of less than 8%. A series of standard dilutions were prepared, ranging from 2,500 ng/L to 40,000 ng/L, by serial dilution of the provided 80,000 ng/L standard. Each well, except for the blank, received 50 µl of streptavidin-HRP. In the standard wells, 50 µl of the appropriate standard concentration was added. In the sample wells, 40 µl of serum and 10 µl of anti-ADMA antibody were added. The plate was then sealed and incubated at 37 °C for one hour. Following incubation, the wells were washed five times using a 25X diluted washing solution in a BioTek ELx50 microplate strip washer. Subsequently, 50 µl each of chromogen A and B were added to all wells, followed by a 10-min incubation at 37 °C. The reaction was stopped with the addition of 50 µl of stop solution, and absorbance readings were taken at 450 nm within 15 min using a BIO-RAD xMark Microplate spectrophotometer. A standard curve was generated by plotting the optical density values of the standards against their known concentrations. The ADMA concentrations of the serum samples were then determined by interpolation from this standard curve.

Statistical analysis was performed on the SPSS for Windows version 18.0 (SPSS Inc. Chicago, IL, USA). The conformity of the data to normal distribution was examined using visual (histogram and probability plots) and analytical methods (Kolmogorov–Smirnov/Shapiro–Wilk tests). Median (1st quartile-3rd quartile) values were used to evaluate numerical data, and frequency distributions and percentages were used to summarize categorical data. Chi-square (χ2) test was used to compare categorical data. The relationship between non-normally distributed numerical data and categorical data was evaluated with the Man-Whitney *U* test. Kruskal–Wallis H test was used for the evaluation of three or more non-normally distributed groups with numerical data. Post-hoc Man-Whitney *U* test and Bonferroni correction were performed for pairwise comparisons between groups with significant Kruskal–Wallis H test results. Correlations of non-normally distributed numerical variables were analyzed with Spearman correlation coefficients. In the evaluation of Spearman correlation coefficients, no correlation below 0.19, low correlation between 0.20–0.39, moderate correlation between 0.40–0.69, high correlation between 0.70–0.89, and very high correlation above 0.90 were accepted. Correlation coefficients with a positive sign indicate that the variables increase or decrease together, while correlation coefficients with a negative sign indicate that one of the variables increases while the other decreases or vice versa. Statistically, *p* < 0.05 was considered statistically significant. The diagnostic decision-making properties of ADMA level in predicting the disease were analyzed by receiver operating characteristics (ROC) curve. In the presence of significant cut-off values, sensitivity, specificity, positive predictive value and negative predictive values of these limits were calculated.

The DeLong test was performed to assess the differences in diagnostic performance between biomarkers. This analysis was performed in Python 3.8 and was computed using the NumPy and SciPy libraries. This nonparametric test was used to compare the areas under the curves (AUC) in ROC analysis and determine whether one biomarker provided significantly better diagnostic accuracy than the other.

This study was conducted with financial support from the Necmettin Erbakan University Faculty of Medicine Scientific Research Project Committee. It was carried out in accordance with the Declaration of Helsinki and Good Clinical Practice guidelines. All participants, or their parents in the case of minors, were informed about the study, and written informed consent was obtained before enrollment. The study protocol was approved by the Necmettin Erbakan University Faculty of Medicine Local Ethics Committee (decision number 2020/2332, dated April 3, 2020).

## Results

Our study included 98 patients with COVID-19 infection, 21 patients diagnosed with MIS-C and 42 cases in the control group. Of the COVID-19 patients, 31% (*n* = 31) were followed as outpatients, 55% (*n* = 55) were hospitalized in the ward, and 12% (*n* = 12) were hospitalized in intensive care unit (ICU). The mean age of the study group was 9.06 ± 5.4 years and the median age was 10 (4.4–13.5) years. Of the total study group, 54.0% (*n* = 87) were boys and 46.0% (*n* = 74) were girls. There was no statistically significant difference between the age and gender distributions of the study and control groups (*p* = 0.088, *p* = 0.730, respectively). The median length of stay of COVID-19 patients hospitalized in the ward was 6.00 days, the median length of stay of patients hospitalized in the ICU was 12.00 days, and the median length of stay of MIS-C patients was 9.00 days.

Comparison of ADMA results of the control group, MIS-C and all COVID-19 patients is shown in Table 1. There was a significant difference in ADMA levels between all groups (*p* < 0.001). The ADMA level of the control group was significantly lower than that of MIS-C and all COVID-19 patients, while the ADMA level of MIS-C patients was significantly higher than that of control and all COVID-19 patients.

**Table 1:** Comparison of ADMA levels across the study groups

	ADMA (ng/L) Median (1st-3rd quartile)	p*	Post Hoc**
Control Group^1^	4081.00 (2526.7–5360.0)	** < **0.001	1 < 3 1,3 < 2,4,5 4 < 5
MIS-C^2^	15,735.5 (13,486.6–20,532.5)		
Outpatient COVID-19^3^	8097.0 (6436.0–10840.0)		
Hospitalized COVID-19^4^	13,195.6 (11,472.4–15,862.2)		
Hospitalized in Intensive Care for COVID-19^5^	19,361.4 (15,596.6–23,367.9)		

^*^Kruskal–Wallis H test **Mann–Whitney *U* test

The median ADMA level of outpatients with a diagnosis of COVID-19 was 8097.0 ng/L (6436.06–10840.0 ng/L), and the median ADMA level of COVID-19 patients hospitalized in the ward was 13,195.60 ng/L (11,472.4–15,862.2 ng/L), The median ADMA level of COVID-19 patients followed up in ICU was 19,361.4 ng/L (15,596.65–23,367.9 ng/L), the median ADMA level of patients followed up with MIS-C was 15,735.50 ng/L (13,486.6–20,532.5 ng/L) and the median ADMA level of control patients was 4081.00 ng/L (2526.7–5360.0 ng/L). There was a significant difference between the study groups in terms of ADMA levels (*p* < 0.001). ADMA levels of the control group were significantly lower than all other groups. Outpatients with a diagnosis of COVID-19 had lower median ADMA levels than patients hospitalized in the ward, patients hospitalized in ICU and MIS-C patients. Those hospitalized in the ward had lower median ADMA levels compared to COVID-19 patients hospitalized in ICU and MIS-C. There was no significant difference between the ADMA levels of COVID-19 and MIS-C patients followed in ICU.

We investigated whether there was a correlation between length of hospitalization and ADMA. In all patient groups, a positive, low-level significant correlation was found between ADMA levels and length of hospitalization (r = 0.289).

**Fig. 1:**
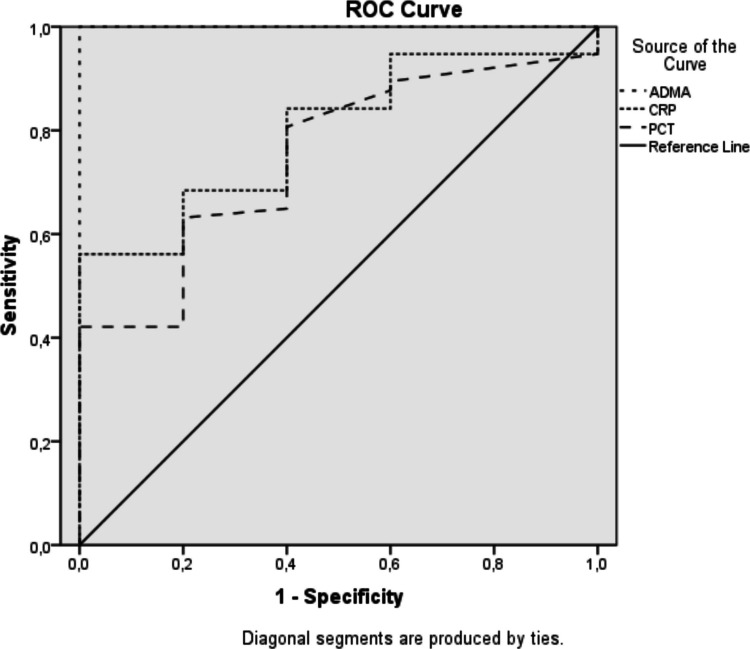
ROC curve plotted for ADMA, CRP and PCT

ROC analysis between patient and control groups for ADMA value showed that when ADMA value was determined as 6135.15 ng/L, sensitivity was 95%, specificity was 100%, positive predictive value was 100% and negative predictive value was 87.5%. In the ROC analysis performed to examine the predictive effect of ADMA levels in the patient and control groups, the p value was statistically significant (*p* = < 0.001) and the AUC was 1.0 (95% Confidence interval: 0.000–1.000). ROC analysis was also performed for C-reactive protein (CRP) and procalcitonin (PCT). The analysis for CRP resulted in *p* = 0.029, AUC 0.796 (95% Confidence interval: 0.642–0.951). The analysis for PCT resulted in *p* = 0.066, AUC 0.749 (95% Confidence interval: 0.561–0.937) (Fig. 1). De Long test was used to compare the AUC of ROC analysis of ADMA, CRP and PCT biomarkers. When ADMA and CRP were compared (p = 0.0098, Z = 2.582), and when ADMA and PCT were compared (*p* = 0.0089, Z = 2.615), significant differences were found in both. In other words, ADMA may be a stronger biomarker in disease discrimination than both CRP and PCT. No significant difference was found when CRP and PCT were compared (*p* = 0.700, Z = 0.378). CRP and PCT are similar in terms of diagnostic performance and these two tests do not have a significant superiority in disease discrimination.

## Discussion

The COVID-19 pandemic has presented a significant global public health challenge. In the pediatric population, one of the most serious complications is MIS-C, typically manifesting 2–6 weeks after initial COVID-19 infection [19]. ADMA, an endogenous inhibitor of NOS, plays a crucial role in vascular health. Elevated ADMA levels disrupt NO production, leading to impaired endothelium-dependent vasodilation [8].

In this study, we compared ADMA levels across different clinical presentations of COVID-19 in children, including MIS-C, and a control group. Our hypothesis was that ADMA, a marker of endothelial dysfunction, would be elevated in patients with COVID-19. We also postulated that ADMA levels would be highest in children with MIS-C, given the severe inflammatory response and cardiac involvement associated with this condition. This could potentially establish ADMA as a valuable indicator of disease severity in pediatric COVID-19 patients.

Our findings demonstrated significant differences in ADMA levels across the groups (*p* < 0.001). The control group, in particular, exhibited markedly lower ADMA levels compared to all other groups, while MIS-C patients had significantly higher levels than all other COVID-19 patient groups. To the best of our knowledge, this is the first investigation examining the relationship between MIS-C and ADMA. Further analysis revealed that outpatients diagnosed with COVID-19 had lower mean ADMA levels than patients hospitalized in the ward, ICU, or those with MIS-C. Additionally, COVID-19 patients in the ward had lower mean ADMA levels than MIS-C and ICU patients. Median ADMA levels in both MIS-C and ICU patients were higher compared to outpatients, ward patients, and the control group. All patient groups individually demonstrated higher ADMA levels than the control group. However, no significant difference in ADMA levels was found between MIS-C and ICU patients. These findings support our initial hypothesis that ADMA levels increase with disease severity, aligning with existing literature highlighting the association between ADMA and conditions such as hypertriglyceridemia, chronic kidney disease, cardiac disease, obesity, diabetes mellitus, and chronic inflammation [20–22].

Our investigation revealed a modest positive correlation between ADMA levels and length of hospitalization (r = 0.289) in the patient group, indicating that higher ADMA values are associated with longer hospital stays. This finding aligns with existing literature [23], suggesting that elevated ADMA levels may reflect increased disease severity and complexity, thus necessitating extended hospitalization.

A limited number of studies in the literature have examined the relationship between ADMA and COVID-19, particularly in pediatric populations. The three identified adult studies consistently reported elevated ADMA levels in patients with severe COVID-19. This present study is the first to investigate ADMA levels in MIS-C, a hyperinflammatory condition with severe organ involvement and potentially poor clinical outcomes. Our results demonstrate significantly higher ADMA levels in the intensive care and MIS-C groups compared to other groups and controls, further supporting the association between increased ADMA and disease severity. We propose that ADMA may be a valuable prognostic marker not only in COVID-19 but also in any disease characterized by endothelial damage. Further research is warranted to explore the potential utility of ADMA in predicting clinical outcomes across a broader range of pathologies.

Previous research has explored the link between endothelial dysfunction and the pathogenesis of viral infections [5–7]. Such connection is attributed to the ability of viruses to disrupt microvascular barrier integrity through a cytokine storm. COVID-19 infection, in particular, is known to cause endothelial damage and contribute to thromboembolic events. An adult study involving 87 patients compared ADMA levels between hospitalized COVID-19 patients requiring intensive care and those who did not. The study found that both ADMA levels and mortality rates were significantly higher in the intensive care group [24].

Compared to adult studies, pediatric research on normal ADMA levels is rather scarce. One study in neonates found that ADMA levels in venous cord blood were approximately 1.06 μM, decreasing to 0.66 μM by postnatal day 2 [25]. In premature infants, plasma ADMA remains stable during the first week but ranges from 0.66–0.95 μM by week 4 [25, 26]. ADMA levels are often higher in childhood than adulthood, decreasing by an average of 15 nM annually from birth to 25 years of age [26]. Data on ADMA cut-off values, particularly in pediatrics, are limited. This study contributes significantly to the literature by providing this valuable data.

Our study has certain limitations. Firstly, it is a single-center study with a relatively small sample size. Secondly, we were unable to identify the specific causative variant at the time of COVID-19 diagnosis, preventing comparisons between variants. Additionally, we could not assess SDMA, another dimethylarginine metabolite, and only measured ADMA once in our patients.

In conclusion, our study is the first to investigate ADMA levels in children with COVID-19 and MIS-C. We observed significantly higher ADMA levels in COVID-19 patients requiring intensive care and in those with MIS-C. We propose that elevated serum ADMA levels may be an indicator of endothelial dysfunction and potential clinical deterioration. Larger, multicenter studies are needed to draw more definitive conclusions on this matter. Although it is not currently widely used, it is a marker with higher sensitivity. Therefore, it can be used in certain cases, especially in diseases where endothelial damage plays role in pathogenesis.

## Data Availability

No datasets were generated or analysed during the current study.

## Abbreviations

ACE 2: Angiotensin-converting enzyme 2
ADMA: Asymmetric dimethylarginine
AUC: Area under the curve
COVID-19: Coronavirus disease 2019
CRP: C-reactive protein
ELISA: Enzyme-linked immunosorbent assay
ICU: Intensive care unit
MIS-C: Multisystem inflammatory syndrome in children
NO: Nitric oxide
NOS: Nitric oxide synthase
PCT: Procalcitonin
SARS-CoV-2: Severe acute respiratory syndrome coronavirus type 2
SDMA: Symmetric dimethylarginine
ROC: Receiver operating characteristics

## References

[1] Ozer MR, Avci A, Baloglu I, Aydogan ZK (2022) Factors associated with intensive care hospitalization in patients with Covid-19. Selcuk Med J 38(2):76–81. 10.30733/std.2022.01551

[2] Suratannon N, Dik WA, Chatchatee P, van Hagen PM (2020) COVID-19 in children: Heterogeneity with in the disease and hypothetical pathogenesis. Asian Pac J Allergy Immunol 38:170–177. 10.12932/ap-170720-0920 32990448 10.12932/AP-170720-0920

[3] Yilmaz D, EkemenKeles Y, Emiroglu M, Duramaz B, Ugur C, Aldemir Kocabas B et al (2023) Evaluation of 601 children with multisystem inflammatory syndrome (Turk MISC study). Eur J Pediatr 182(12):5531–5542. 10.1007/s00431-023-05207-6 37782350 10.1007/s00431-023-05207-6

[4] Cai H, Harrison DG (2000) Endothelial Dysfunction in cardiovascular diseases: The role of oxidant stress. Circ Res 87(10):840–844. 10.1161/01.res.87.10.840 11073878 10.1161/01.res.87.10.840

[5] Krautkrämer E, Zeier M, Plyusnin A (2013) Hantavirus Infection: An emerging infectious disease causing acute renal failure. Kidney Int 83(1):23–27. 10.1038/ki.2012.360 23151954 10.1038/ki.2012.360

[6] Mazzuca P, Caruso A, Caccuri F (2016) HIV-1 infection, microenvironment and endothelial cell dysfunction. New Microbiol 39(3):163–173 27704142

[7] Ismail A, Mahboob T, Raju CS, Sekaran SD (2019) Zika Virus Modulates Blood-brain barrier of brain microvascular endothelial cells. Trop Biomed 36(4):888–897 33597462

[8] Sibal L, Agarwal SC, Home PD, Boger RH (2010) The role of asymmetric dimethylarginine (ADMA) in endothelial dysfunction and cardiovascular disease. Curr Cardiol Rev 6(2):82–90. 10.2174/157340310791162659 21532773 10.2174/157340310791162659 PMC2892080

[9] Blackwell S (2010) The biochemistry, measurement and current clinical significance of asymmetric dimethylarginine. Ann Clin Biochem 47(1):17–28. 10.1258/acb.2009.009196 19940201 10.1258/acb.2009.009196

[10] Zha D, Fu M, Qian Y (2022) Vascular endothelial glycocalyx damage and potential targeted therapy in COVID-19. Cells 11(12):1972. 10.3390/cells11121972 35741101 10.3390/cells11121972 PMC9221624

[11] Bode-Böger SM, Scalera F, Kielstein JT, Martins-Lobenhoffer J, Breithardt G, Fobker M, Reinecke H (2006) Symmetric Dimethylarginine: A new combined parameter for renal function and extent of coronary artery disease. J Am Soc Nephrol 17(4):1128–1134. 10.1681/asn.2005101119 16481412 10.1681/ASN.2005101119

[12] Schepers E, Barreto DV, Liabeuf S, Glorieux G, Eloot S, Barreto FC, Massy Z, Vanholder R (2011) Symmetric dimethylarginine as a proinflammatory agent in chronic kidney disease. Clin J Am Soc Nephrol 6(10):2374–2383. 10.2215/CJN.01720211 21817129 10.2215/CJN.01720211 PMC3359555

[13] Huang LT, Hsieh CS, Chang KA, Tain YL (2012) Roles of nitric oxide and asymmetric dimethylarginine in pregnancy and fetal programming. MDPI 13(11):14606–14622. 10.3390/ijms131114606 10.3390/ijms131114606 PMC3509599 23203083

[14] Park MJ, Oh KS, Nho JH, Kim GY, Kim D (2016) Asymmetric dimethylarginine (ADMA) treatment induces apoptosis in cultured rat mesangial cells via endoplasmic reticulum stress activation. Cell Biol Int 40(6):662–670. 10.1002/cbin.10602 26992443 10.1002/cbin.10602

[15] Shirakawa T, Kako K, Shimada T, Nagashima Y, Nakamura A, Ishida J, Fukamizu A (2011) Production of Free methyl arginines via the proteasome and autophagy pathways in cultured cells. Mol Med Rep 4(4):615–620. 10.3892/mmr.2011.488 21584492 10.3892/mmr.2011.488

[16] Pekarova M, Kubala L, Martiskova H, Bino L, Twarogova M, Klinke A et al (2013) Asymmetric dimethylarginine regulates the lipopolysaccharide-induced nitric oxide production in macrophages by suppressing the activation of NF-kappaB and iNOS expression. Eur J Pharmacol 713(1–3):68–77. 10.1016/j.ejphar.2013.05.001 23665490 10.1016/j.ejphar.2013.05.001

[17] Tain YL, Hsu CN (2017) Toxic dimethyl arginines: asymmetric dimethylarginine (ADMA) and symmetric dimethylarginine (SDMA). Toxins (Basel) 9(3):92. 10.3390/toxins9030092 28272322 10.3390/toxins9030092 PMC5371847

[18] CDC (2020) Pediatric inflammatory multisystem syndrome and SARS-CoV-2 infection in children. https://www.ecdc.europa.eu. Accessed 15 May 2020

[19] Patel JM (2022) Multisystem inflammatory syndrome in children (MIS-C). Curr Allergy Asthma Rep 22(5):53–60. 10.1007/s11882-022-01031-4 35314921 10.1007/s11882-022-01031-4 PMC8938222

[20] Zoccali C, Bode-Böger S, Mallamaci F, Benedetto F, Tripepi G, Malatino L et al (2001) Plasma concentration of asymmetrical dimethylarginine and mortality in patients with end-stage renal disease: a prospective study. Lancet 358:2113–2117. 10.1016/s0140-6736(01)07217-8 11784625 10.1016/s0140-6736(01)07217-8

[21] Schnabel R, Blankenberg S, Lubos E, Lackner KJ, Rupprecht HJ, Espinola-Klein C et al (2005) Asymmetric dimethylarginine and the risk of cardiovascular events and death in patients with coronary artery disease: Results from the athero gene study. Circ Res 97(5):e53–59. 10.1161/01.res.0000181286.44222.61 16100045 10.1161/01.RES.0000181286.44222.61

[22] Winkler MS, Nierhaus A, Rösler G, Lezius S, Harlandt O, Schwedhelm E, Böger RH, Kluge S (2018) Symmetrical (SDMA) and asymmetrical dimethylarginine (ADMA) in sepsis: high plasma levels as combined risk markers for sepsis survival. Crit Care 19(22):216. 10.1186/s13054-018-2090-1 10.1186/s13054-018-2090-1 PMC6145330 30231905

[23] Hannemann J, Balfanz P, Schwedhelm E, Hartmann B, Ule J, Müller-Wieland D et al (2021) Elevated serum SDMA and ADMA at hospital admission predict in-hospital mortality of COVID-19 patients. Sci Rep 11(1):9895. 10.1038/s41598-021-89180-w 33972591 10.1038/s41598-021-89180-w PMC8110746

[24] Karacaer C, Yaylaci S, Demirci T, Cekic D, Suner KO, Cokluk E, Varim C (2022) Association of mortality and endothelial dysfunction with serum ADMA level in COVID-19 patients. Pak J Med Sci 38(7):1808–1815. 10.12669/pjms.38.7.5327 36246680 10.12669/pjms.38.7.5327 PMC9532643

[25] Vida G, Sulyok E, Ertl T, Martens-Lobenhoffer J, Bode-Boger SM (2007) Plasma asymmetric dimethylarginine concentration during the perinatal period. Neonatology 92:8–13. 10.1159/000098411 17596731 10.1159/000098411

[26] Lücke T, Kanzelmeyer N, Kemper MJ, Tsikas D, Das AM (2007) Developmental changes in the Larginine/nitric oxide pathway from infancy to adulthood: plasma asymmetric dimethylarginine levels decrease with age. Clin Chem Lab Med 45(11):1525–1530. 10.1515/cclm.2007.300 17892438 10.1515/CCLM.2007.300

